# Management of Hepatic Sarcoidosis: A Retrospective Analysis of Patients at a University Hospital

**DOI:** 10.31138/mjr.111124.thr

**Published:** 2025-07-17

**Authors:** Manush Sondhi, Sulman Hasan, Kavya Vadlamudi, Mohammad Alfrad Nobel Bhuiyan, Anusheh Ali, Tabitha Muutu, Samina Hayat, Sarwat Umer, Kinza Muzaffar

**Affiliations:** 1 Department of Internal Medicine, Louisiana State University, Shreveport, Louisiana, United States of America;; 2 Department of Rheumatology, Louisiana State University, Shreveport, Louisiana, United States of America;; 3 Louisiana State University, Shreveport, Louisiana, United States of America

**Keywords:** sarcoidosis, hepatitis, methotrexate, azathioprine, alkaline phosphatase

## Abstract

**Objective:**

To explore efficacy of medications, namely steroids, ursodeoxycholic acid (UDC), methotrexate (MTX), azathioprine, mycophenolate mofetil (MMF), and infliximab in the treatment of hepatic sarcoidosis (HS).

**Methods:**

We searched for the patients using ICD codes for sarcoidosis (ICD-10: D86) and granulomatous hepatitis (ICD-10: K75.3) at Louisiana State University, Shreveport, and generated 150 unique medical record numbers. We retrospectively reviewed notes, labs, imaging, and medications, and used descriptive statistics to calculate percentages.

**Results:**

47 patients had a diagnosis of HS. 72% of patients had ALP elevation of >200. 36 (76%) patients received steroids, 20 (42%) had MTX, 5 (10%) had azathioprine, 5 (10%) had rituximab, 12 (25%) had infliximab, 3 (6%) had UDC, 21 (44%) had MMF. 12 patients received a combination of prednisone with either MTX, azathioprine, MMF, infliximab, or rituximab. Treatment response was measured based on ALP improvement. 55% of patients responded to prednisone, 45% to MTX, 40% to azathioprine, 60% to rituximab, 66% to infliximab, 47% to MMF, and 30% to UDC.

**Conclusion:**

Majority of the patients presented with ALP elevation of >200. Liver biopsy was performed in only 27% of the patients. Despite being one of the recommended initial therapies, UDC was used in only 3% of patients. Steroids were most commonly used. Among all the steroid-sparing agents, infliximab showed the best efficacy. Similarly, MTX showed improvement, but it was generally avoided due to the risk of hepatotoxicity. Azathioprine, MMF, and rituximab were used either in combination or as sole therapies and have shown improvement in ALP.

## INTRODUCTION

Sarcoidosis is characterised by the presence of non-ca-seating granulomas, and it typically affects the lungs. However, in about 5–10% of cases, it can involve the gastrointestinal system, and hepatic sarcoidosis (HS) can occur in 11–80% of those cases.^1^ HS can manifest with various symptoms or may be asymptomatic and discovered incidentally during imaging or laboratory tests. The clinical presentation of HS can vary, ranging from asymptomatic liver enzyme elevation to right upper quadrant pain, jaundice, and pruritus. Identifying HS poses a formidable challenge, typically demanding a fusion of clinical scrutiny, diagnostic imaging modalities (including ultrasound, CT scans, or MRI), and assessments of liver functionality. Elevated alkaline phosphatase (ALP) is considered the most reliable indicator of liver involvement. A liver biopsy may be necessary to confirm the presence of granulomas in the liver and rule out other liver diseases. Biomarkers and imaging studies are not highly sensitive or specific for HS, which makes liver biopsy the most definitive diagnostic tool. Liver biopsy commonly unveils non-caseating granulomas characterised by central macrophages encircled by a periphery of diverse inflammatory cells. It is crucial to differentiate HS from other liver diseases, particularly primary biliary cirrhosis, and primary sclerosing cholangitis. The treatment for HS depends on the severity of liver involvement and the presence of symptoms. In instances of mild severity, intervention may not be imperative, and the condition can be effectively handled through vigilant observation and routine monitoring. However, most studies support the use of steroids, ursodeoxycholic acid (UDC), methotrexate (MTX), azathioprine, mycophenolate mofetil (MMF), and infliximab in the treatment of HS, although evidence-based guidelines for HS treatment are lacking. The goal of treatment is to prevent the development of portal hypertension and cirrhosis, which are associated with long-standing hepatic sarcoidosis. In this study, we aim to explore the efficacy of the abovementioned medications in treating HS using a retrospective analysis of medical records from patients with HS at our institution.

## METHODS

We conducted our study as a retrospective analysis, approved by the institutional review board at Louisiana State University (LSU), Shreveport. We identified 150 unique medical record numbers by searching for patients with relevant ICD-10 codes for both sarcoidosis (ICD-10: D86) and granulomatous hepatitis (ICD-10: K75.3) in the electronic medical records (EMR). We thoroughly reviewed the notes, laboratory results, liver biopsy reports, imaging findings, and medication records available in the EMR. Descriptive statistics were employed to calculate percentages, and open-source software was used for data analysis. The analysis involved a comprehensive review of the medical records in extracting relevant information related to sarcoidosis and granulomatous hepatitis to better understand the clinical characteristics, demographics, and other pertinent details of the patients included in the study.

## RESULTS

Our study identified 47 patients diagnosed with hepatic sarcoidosis. Among them, 78% were females aged 20–40. 93% were African Americans, indicating a higher prevalence of HS in this demographic group.

We observed that 25% of patients had sole liver involvement, while 75% had multiorgan involvement, suggesting that HS can often present as a systemic disease. Elevated ALP levels (>200) were found in 72% of patients, indicating liver dysfunction. A liver ultrasound was performed in 48% of patients, and liver biopsies were performed in 27% of patients to confirm the diagnosis.

In terms of treatment, 36 patients (76%) received steroids with or without steroid-sparing agents, 20 patients (42%) were treated with MTX, 5 patients (10%) received azathioprine, 5 patients (10%) were treated with rituximab, 12 patients (25%) received infliximab, 3 patients (6%) received UDC, and 21 patients (44%) received MMF. Additionally, 12 patients received a combination of prednisone with either MTX, azathioprine, MMF, infliximab, or rituximab.

We evaluated treatment response based on ALP improvement. Among the patients treated with specific medications, 55% responded to prednisone, 45% to MTX, 40% to azathioprine, 60% to rituximab, 66% to infliximab, 47% to MMF, and 30% to UDC. These findings suggest varying degrees of treatment effectiveness for different medications in managing hepatic sarcoidosis in this patient population.

Overall, our study provides insights into the demographic characteristics, clinical presentation, and treatment approaches for patients with hepatic sarcoidosis based on a retrospective analysis of medical records at LSU, Shreveport.

## DISCUSSION

Sarcoidosis is a condition distinguished by the presence of clusters of specialised immune cells called epithelioid granulomas. These granulomas are usually non-caseating and are not caused by tuberculosis, fungal infections, cancer, or other conditions that commonly trigger similar immune responses.^2^ The prevalence of hepatic sarcoidosis is greatest between 45–66 years of age and in African Americans more than Caucasians, with approximately twice the frequency.^3^ In our study, liver involvement was primarily seen in patients with multisystem sarcoidosis.

Hepatic sarcoidosis is primarily asymptomatic, even in the setting of liver function tests. Rarely, it can manifest with non-specific symptoms such as fever, weight loss, night sweats, malaise, and fatigue, presumably from the systemic nature of the disease rather than specific organ involvement.^4^ In a retrospective study, it was observed that fatigue emerged as the predominant initial symptom of hepatic sarcoidosis, followed by pruritus, weight loss, and hepatomegaly.^5^

Normal ALP values in adults typically range from 30 to 120 IU/L, with abnormal levels considered as 2–3 times the upper limit. In individuals showing signs and symptoms suggestive of sarcoidosis affecting the liver, ALP may be significantly increased, reaching 5–10 times the upper limit of normal. On the other hand, elevations in aminotransferase levels are typically milder and occur less frequently compared to the elevations seen in ALP levels.^6^ A population-based study found that 88% of hepatic sarcoidosis patients had elevated ALP, while elevated transaminases were less frequent.^7,8^ In our study, too, the majority of the patients presented with an ALP elevation of >200. We defined the normalisation of ALP levels as an indicator of treatment response. Infrequent occurrences of hyperbilirubinemia and hypoalbuminemia are typically associated with cirrhosis in cases of hepatic sarcoidosis. Serum angiotensin-converting enzyme (ACE) levels, commonly used as a marker for sarcoidosis, may be elevated in 60% of patients with active disease but lack sensitivity and specificity (positive predictive value 84%, negative predictive value 74%), with elevated ACE indicating a more severe course and increased corticosteroid need.^9^ Studdy et al. also showed that steroid therapy significantly reduced serum ACE activity, highlighting the connection between systemic inflammation and treatment response.^10^

Asymptomatic hepatic lesions may be initially detected on chest CT. Abdominal CT scans typically reveal hepatomegaly and multiple nodular lesions with decreased density ranging in size from 1 mm to 3 cm.^11^ On MRI, these nodules appear hypointense on T1-weighted images and hyperintense on T2-weighted images compared to the surrounding liver tissue. These lesions are often found around the portal veins.^12^

A liver biopsy is recommended for patients with moderate or severe abnormalities in liver tests, such as levels exceeding three times the upper limit of normal. Biopsy specimens more remarkable than 2 cm in length typically show non-necrotising granulomas. However, since other conditions can also cause granulomatous lesions in the liver, it is vital to identify characteristic extrahepatic manifestations of sarcoidosis, examine the location and appearance of the granulomas within the liver, conduct special stains or cultures to rule out infectious organisms and exclude malignancy or drug-induced granulomas. Liver biopsies of patients with sarcoidosis reveal the presence of granulomas in about 50 to 65 percent of cases. However, symptomatic hepatic sarcoidosis, where the condition causes noticeable symptoms, occurs in a smaller percentage, ranging from 5 to 15 percent of patients.^13^ In our study, liver biopsy was performed in only 27% of the patients, further emphasising the need to rule out the other causes of ALP elevation and granulomatous liver changes. Hepatic sarcoidosis in the absence of clinical or biochemical derangements does not require treatment. However, if patients have symptoms of liver disease or are at high risk for liver complications, medication-based treatment should be considered.

**Table 1.** Demographic breakdown of the patients in the study.

**Characteristics**	**Sample (n)**
**Sex**	
Male	10
Female	37
**Ethnicity**	
African American	44
Caucasian	3
**Age**	
>60 years	11
<60 years	36
**AST, ALT**	
Elevated	28
Not Elevated	19
**Extra-Hepatic Involvement**	
Lung	19
Eye	2
Skin	6
Lymph Node	14
CNS	1
**Therapy Type**	
Steroids	36
Steroids + another drug	32

UDC can be the consensus first-line treatment for hepatic sarcoidosis in the presence of cholestatic injury.^4^ It defers the advancement of the condition by reducing the biliary secretion of cholic and chenodeoxycholic acid while also impeding the intestinal absorption of bile salt. Despite being one of the recommended initial therapies for HS, UDC was used in only 3% of patients in our study.

Corticosteroids have the ability to diminish hepatic granuloma count by dampening the inflammatory reaction and decreasing liver size. Steroids were most commonly used in our study. Although steroids may normalise liver function tests and reduce symptoms, they may not prevent disease progression on serial biopsies. In a study involving 63 patients with hepatic sarcoidosis treated with prednisone, around one-third of the patients experienced a complete clinical response, another one-third had a partial response, and the remaining one-third did not respond to the treatment.^14^ The SARCORT trial found that high-dose prednisolone (40 mg/day) was no more effective than low-dose prednisolone (20 mg/day) in improving outcomes or quality of life in sarcoidosis, suggesting higher doses offer no additional benefit.^15^

Among all the steroid-sparing agents, infliximab showed the best efficacy. Infliximab infusion given every 4–6 weeks in refractory cases had resulted in disease remission. Our experience with infliximab indicates that multiple sequential infusions are needed for disease control, and once the infusion is stopped, patients may relapse. The effectiveness of infliximab lies in its ability to suppress the granulomatous response.^16^ Similarly, MTX showed improvement, but the drug appears to take 6 months to become effective, limiting its use in acute settings. In our study, it was avoided in new patients due to the risk of hepatotoxicity and the need for frequent lab testing, but it was continued in those who had been on it prior to the study. Azathioprine, MMF, and rituximab were used either in combination or as sole therapies and have shown improvement in ALP. There was no significant difference in ALP resolution between patients with isolated liver involvement and those with systemic granulomatous inflammation. Also, there was no clear evidence of a significant difference in outcomes between monotherapy with steroids and combination therapy with steroids and DMARDs/biologics. Treatment was tailored to the individual, taking into account patient response and tolerance. Combination therapy has been widely used and found to be helpful in the treatment of chronic and refractory sarcoidosis. However, care must be taken to monitor for increased toxicity. Some of these medications have limitations based on the side effects and insurance coverage.^1^ A small percentage of patients with hepatic sarcoidosis may develop cirrhosis (6 %), a cholestatic liver disease characterised by diffuse biliary strictures that resemble sclerosing cholangitis, obstructive jaundice caused by lymphadenopathy in the hepatic hilum, or hepatic vein thrombosis.^10^ Approximately 3% of patients with hepatic sarcoidosis may develop portal hypertension, which can be caused by biliary fibrosis, cirrhosis, or in rare cases, periportal granulomas that restrict blood flow in the portal vein.^10,17^ Liver transplantation may be considered in rare cases of hepatic vein thrombosis caused by the compression of hepatic veins by sarcoid granulomas.^10^

**Figure 1.**
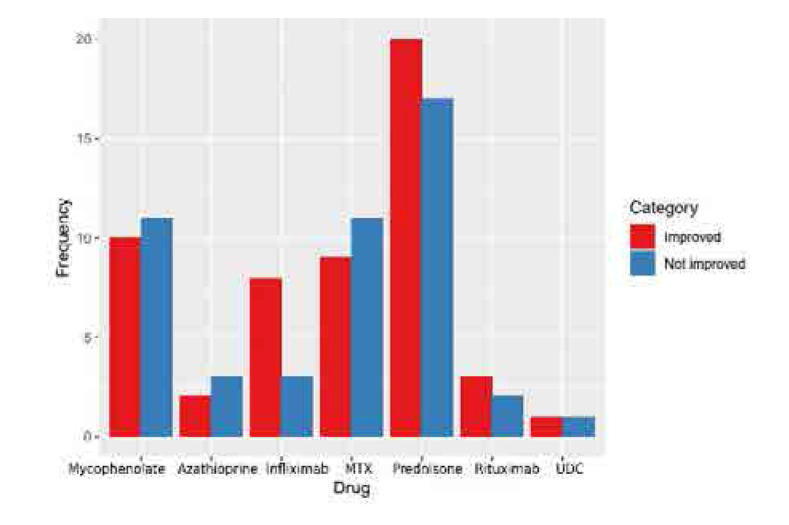
Graph depicting a comparison of the drugs used in the study for the treatment of hepatic sarcoidosis.

Hepatic sarcoidosis is managed by regularly checking the levels of serum alkaline phosphatase and transaminases in the blood. The specific frequency of these tests is not standardised and depends on the patient’s clinical response to treatment. In sarcoidosis, fatalities typically result from severe pulmonary, cardiac, or central nervous system complications, rather than involvement of the liver. Still, given the development of severe complications like cirrhosis and portal hypertension, it is crucial to recognise the liver involvement early in the disease course using the appropriate diagnostic approach. In order to ascertain the effectiveness of steroid-sparing medications in preventing the advancement of liver disease, it is essential to conduct randomised controlled trials.

## CONFLICT OF INTEREST

The authors declare no conflict of interest.
